# Trends in BMI of Indonesian adults between 1993 and 2014: a longitudinal population-based study

**DOI:** 10.1017/S1368980023000472

**Published:** 2023-07

**Authors:** Tri Nisa Widyastuti, Robin Turner, Helen Harcombe, Rachael McLean

**Affiliations:** 1 Department of Preventive and Social Medicine, Dunedin School of Medicine, University of Otago, 18 Frederick Street, Dunedin 9016, New Zealand; 2 Biostatistics Centre, Division of Health Sciences, University of Otago, 18 Frederick Street, Dunedin 9016, New Zealand

**Keywords:** BMI trajectory, Adult, Obesity, Indonesia

## Abstract

**Objective:**

To examine the trajectories of BMI in Indonesian adults from 1993 to 2014, investigating different patterns by sex and birth cohort.

**Design:**

Longitudinal study: secondary data analysis of the Indonesian Family Life Survey, a large-scale population-based longitudinal study, had their height and weight measured up to five times throughout the 21-year study period (1993–2014). The change in BMI across time was estimated using group-based trajectory models, then differences by sex and birth cohort were investigated using random effect (mixed) models.

**Setting:**

Thirteen out of twenty-seven provinces in Indonesia.

**Participants:**

Indonesian adults aged 19 years and older (*n* 42 537) were included in the analysis.

**Results:**

Mean BMI in adults increased between 1993 (21·4 kg/m^2^) and 2014 (23·5 kg/m^2^). The group-based trajectory model found three distinct groups with mean BMI increasing more rapidly in the most recent time periods. The first group (56·7 % of participants) had a mean BMI entirely within the normal weight range; the second group (34·7 %) started in the normal weight category and were obese, on average by the end of the study period; and the third group (8·6 %) were always in the obese category, on average. The shape of these three trajectories differed by gender (*P* < 0·001) and birth cohort (*P* < 0·001).

**Conclusions:**

The mean BMI among Indonesian adults has increased between 1993 and 2014, driven by those in the most recent birth cohorts. Our findings support the urgent need for targeted overweight and obesity prevention and intervention programmes in Indonesia.

The Global Burden of Disease study shows mean BMI has been increasing since the latter half of the 20th century in many countries^(1)^. In middle-income countries, this increase has happened much more recently than in high-income countries^(2,3)^. This rapid increase in mean BMI has been associated with increases in a number of non-communicable diseases including CVD, diabetes and some cancers^(1,4)^. Data collected worldwide shows that global adult obesity prevalence has increased from an estimated 29 % to 37 % in men and 30 % to 38 % in women over the last 30 years. Increases have been particularly rapid in many low- and middle-income countries, such as in Pacific Island countries and the Caribbean, Middle East and Central America^(5,6)^.

Several repeated cross-sectional studies using the Indonesian Family Life Survey (IFLS) have reported an increasing trend in mean BMI or overweight/obesity prevalence for adults in Indonesia^(7–9)^. The National Report of Indonesia Basic Health Research conducted by the Indonesia Ministry of Health reported a substantial increase in the prevalence of overweight and obesity among the adult population from 8·8 % to 13·6 % and 10·3 % to 21·8 %, respectively, from 2007 to 2018^(10–14)^. These studies were based on cross-sectional designs, so they are limited to estimating prevalence and cannot track individual weight change over time. Development of nuanced obesity prevention/intervention programmes and policies requires a profound understanding of under-recognised characteristics of BMI trends^(8)^.

Crucial questions such as whether the rising trends in mean BMI might be different between males and females and between birth cohorts, which could identify high-risk population groups and lead to more targeted prevention and interventions, have not yet been addressed for the Indonesian population. To fill this evidence gap, we aimed to examine the trajectories of BMI in Indonesian adults from 1993 to 2014, with specific objectives to examine whether the pattern of mean BMI differs by sex and birth cohort.

## Methods

### Study design and data sources

We used publicly available (https://www.rand.org/well-being/social-and-behavioral-policy/data/FLS/IFLS.html) ^(15)^ data from the Indonesian Family Life Survey (IFLS), a large population-based longitudinal study conducted over a 21-year period (1993–2014). The survey and sampling methods have been described in detail elsewhere^(16–19)^. In brief, there are five waves of data available from 1993, 1997, 2000, 2007 to 2014. The initial sampling frame included randomly selected households from thirteen out of twenty-seven provinces in Indonesia, representing around 83 % of the Indonesian population in 1993. The initial households along with their ‘split-off’ (new additional family member/s due to marriage or new birth) were followed in the surveys conducted in waves 2 to 5. The drop-out rate was considered low (less than 10 %) in all follow-up surveys^(19)^.

We included all adults aged 19 years and older. Individuals with missing values on age or sex, where these were unable to be determined from any wave, were excluded. Pregnant women and individuals with implausible values of height (less than 100 cm or more than 200 cm) and weight (< 25 kg or > 200 kg) were also excluded from the analysis^(8)^.

The outcome variable for this study was BMI calculated from weight in kilograms (kg) divided by height in meters squared (m^2^). Height and weight were measured by trained health personnel such as nurses, midwives or medical doctors^(16)^. BMI was analysed as a continuous variable. The WHO criteria for Asian populations BMI cut-off points were used as a reference in the graphs. The WHO BMI for Asian population defines BMI < 18·5 kg/m^2^ as underweight; 18·5–22·9 kg/m^2^ as normal weight; 23–24·9 kg/m^2^ as overweight and > 25 kg/m^2^ as obese^(20)^.

The explanatory variables were sex and birth cohort/age. Sex was treated as dummy variable as 1 if male and 0 if female. Age was measured in years from the difference in years between self-reported date of birth and the date of measurement (or self-reported age if participants did not know their date of birth) and then categorised in either 5-year or 10-year intervals depending on the level of detail required for the analysis. Birth cohort was classified into a decade of birth and further grouped into 1930s and earlier; 1940s; 1950s; 1960s and 1970–1990s.

### Statistical analysis

The characteristics of the sample were summarised using number and percentage for sex, age and birth cohort using the first measure per person (regardless of wave). We initially summarised BMI with mean and sd for each wave (by sex) and by plotting the distribution of BMI for each of the five survey waves both overall and by sex, using a kernel density smoother.

To investigate the changing pattern of BMI across time, by age group and birth cohort, we plotted: (a) mean BMI by age group for different periods, observations within each period connected; (b) mean BMI by period for different age groups, observations within each age-group connected; (c) mean BMI by age for different birth cohort, observations within each birth cohort connected and (d) mean BMI by birth cohort for different age groups, observations within each age-group connected.

We used group-based trajectory model (a finite mixture model) to estimate the shape and number of BMI trajectory groups using a censored normal distribution. The number of groups was decided based on having the largest Bayesian information criterion, and the shape was based on the highest-order polynomial parameter where the *P* value was less than 0·05. We also constrained the groups to have more than 5 % of the sample assigned to them to avoid a non-parsimonious final model^(21)^. The adequacy of the final model was assessed using diagnostic procedures: the assignment of the average posterior probabilities for each group should be 0·7 or higher; the odds of correct classification for each group should be 0·5 or higher and the proportion of the observed probability for each group should be similar with the proportion of estimated from the model^(22)^.

Mixed models, with random effects to account for the within-person correlation, were used to test if the identified trajectory groups were different by sex and birth cohort^(23,24)^. We conducted a sensitivity analysis where we excluded participants who had one measure only as they could not contribute information about the within-person change. All analysis was conducted in Stata version 17·0^(25,26)^.

## Results

**Fig. 1:**
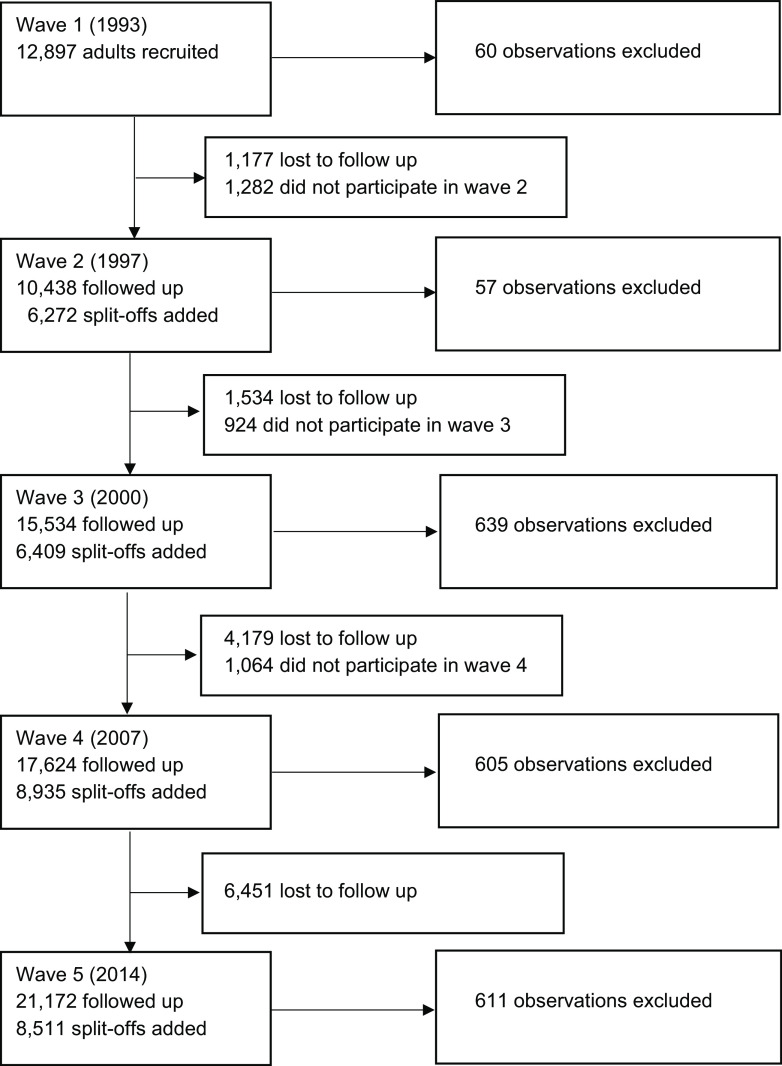
Flow diagram of exclusion criteria and total sample

There were 105 820 observations from 42 537 individuals included in the final analysis after exclusion criteria were applied. Figure 1 shows the recruitment and exclusions across the study waves. The description of the included participants at first observation is presented in Table 1. The majority of participants (52·8%) were aged 19–29 years when they first participated in the survey, followed by those aged 30–39 years (22·9%), 40–49 years (10%), 50–59 years (7·1%), 60–69 years (4·3%) and > 70 years (2·9%). The proportion of males and females was similar (48·8 % males, 51·2% females). A higher proportion of participants completed two or more surveys (65·5%) than those who only completed one survey (34·5%).

**Table 1:** Characteristics of the Indonesian adult participants at their first observation

	*n*	%
Total	42 537	100
Age at recruitment (years)		
19–29	22 478	52·8
30–39	9722	22·9
40–49	4264	10·0
50–59	3004	7·1
60–69	1843	4·3
70+	1226	2·9
Sex		
Male	20 778	48·8
Female	21 759	51·2
Number of waves completed		
1	14 681	34·5
2	9761	23·0
3	6500	15·2
4	5858	13·8
5	5737	13·5

Mean BMI increased from 21·0 kg/m^2^ in 1993 (sd 3·0) to 22·5 kg/m^2^ (sd 3·9) in males and from 21·7 kg/m^2^ (sd 3·7) in 1993 to 24·4 kg/m^2^ (sd 4·8) in 2014 in females (online Supplementary Material).

**Fig. 2:**
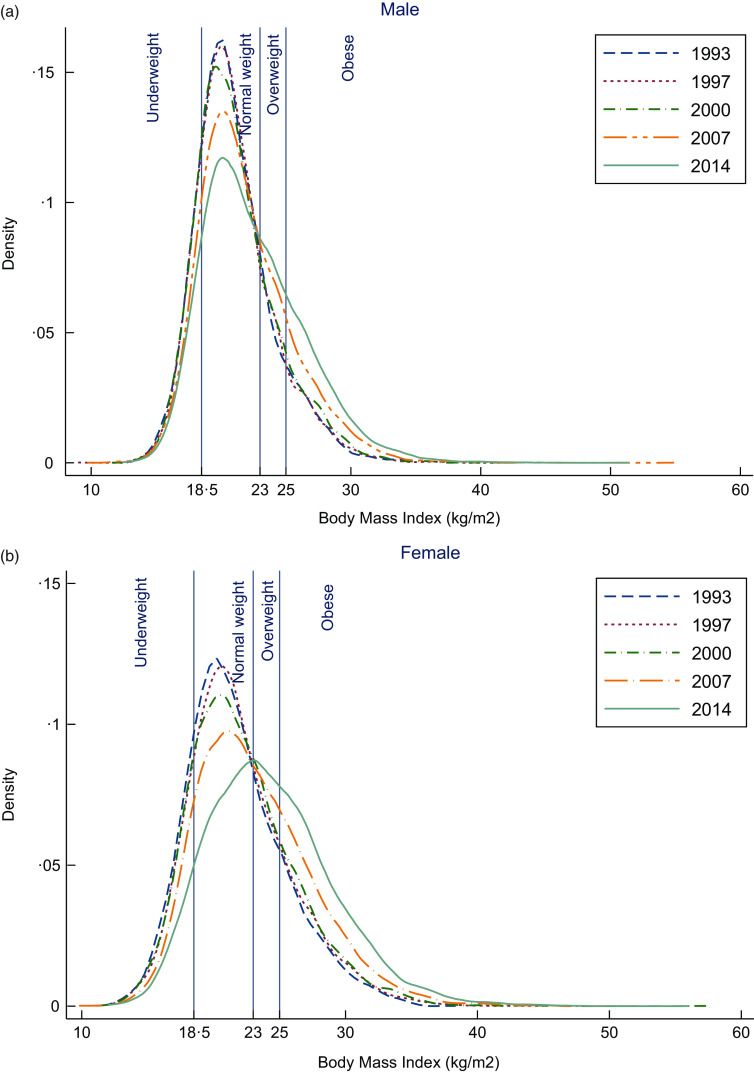
The distribution of BMI in Indonesian adults, 1993–2014: (a) in males, (b) in females

The distribution of BMI changed over time from 1993 to 2014 both in males and females (Fig. 2), showing that the proportion of participants within the overweight and obese category has increased with time, more so for females than males.

**Fig. 3:**
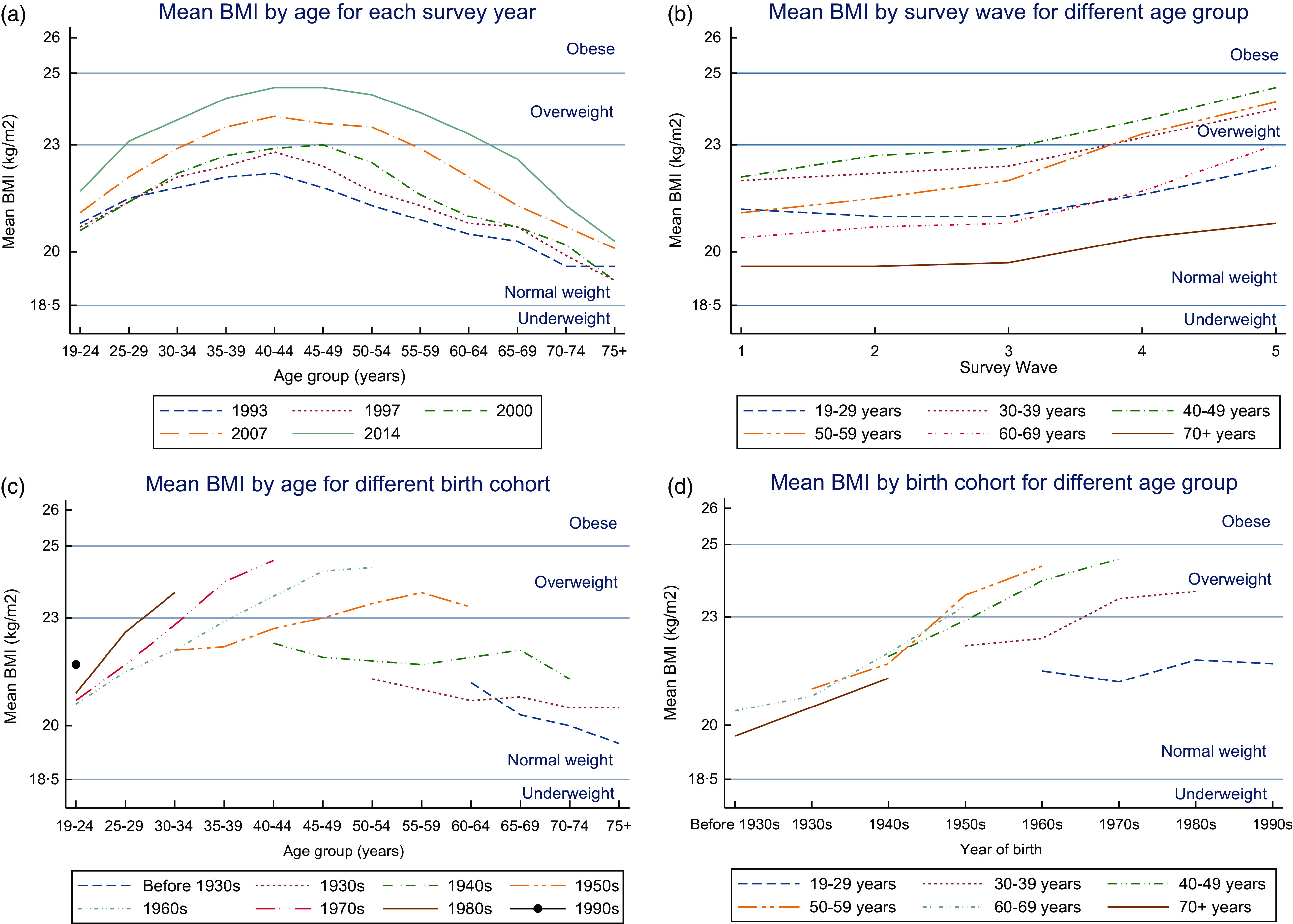
Mean BMI of adults: (a): Mean BMI of adults by age for each survey year. (b): Mean BMI of adults by year of survey for different age groups. (c): Mean BMI of adults by age group for different birth cohort. (d): Mean BMI of adults by birth cohort for different age groups

Figure 3 shows the mean BMI by age, period of survey and birth cohort. Plot (a) shows the mean BMI by age for each survey wave (period). Mean BMI increased with age then declined with the peak in the 40–44-year age group for each survey wave. Mean BMI increased with each survey wave (1993–2014). Plot (b) shows mean BMI by a wave of survey (period) for different age groups. Mean BMI increased over time from 1993 to 2014 among all age groups. The exception is that mean BMI slightly decreased between 1997 and 2007 and substantially increased after 2007 among the 19–29-year age group. Mean BMI was highest among those aged 40–49 years overall. Plot (c) illustrates mean BMI by age for different birth cohorts. Mean BMI changed with age and the birth cohort. Higher mean BMI was present for those born in the recent decades than the mean BMI of those born in the earlier decades. For instance, those born in 1970s had substantially higher mean BMI (24·6 kg/m^2^) when they were aged 40–44 years than those born in 1940s when they were at the same age (22·3 kg/m^2^). More recent birth cohorts also have steeper increases in BMI over time, with the earliest birth cohorts showing a decrease in BMI over time. Plot (d) showing mean BMI by birth cohort for different age groups, confirms the illustration shown in plot c that Indonesian adults born in the recent decades had higher mean BMI than those born in earlier decades when they were the same age.

**Fig. 4:**
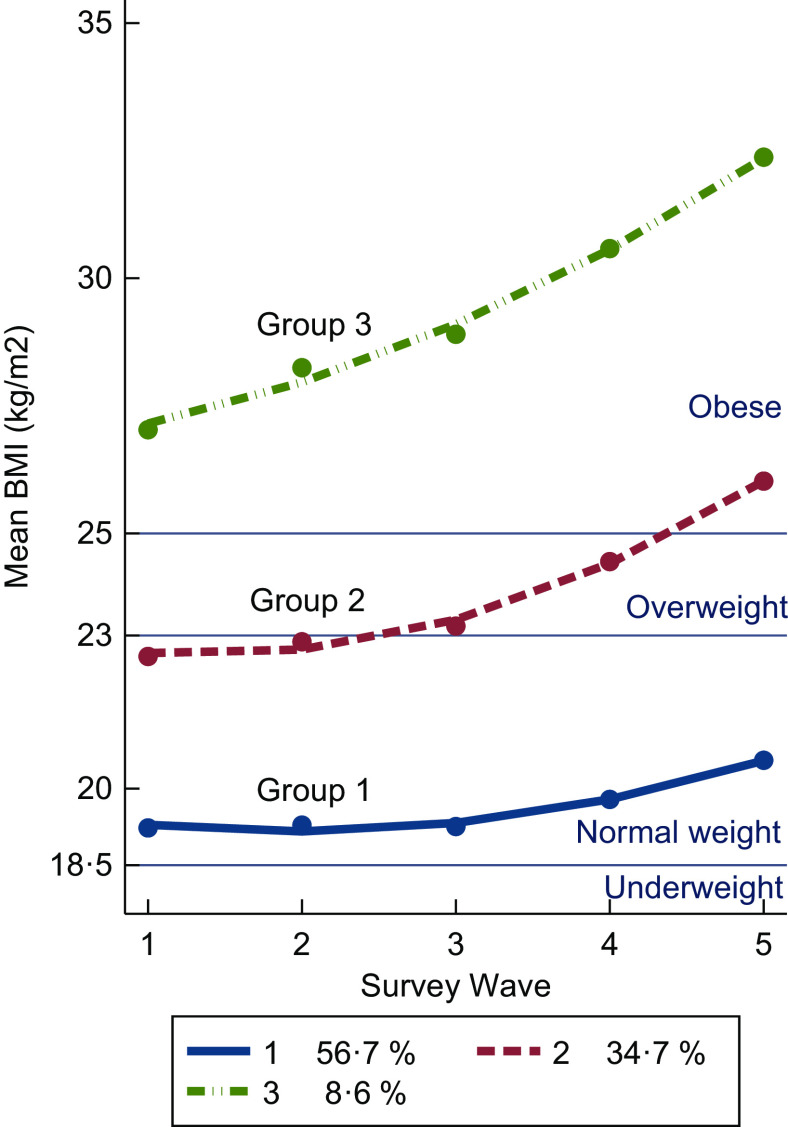
Estimated BMI trajectories (solid lines) of adults from the group-based trajectory model by survey wave with observed group means at each survey year (dot), with the actual group percentages in the figure legend

Using the group-based trajectory model, we identified three distinct trajectory groups among Indonesian adults (Fig. 4). Each trajectory is quadratic in shape (with an increasing slope in more recent waves). Group 1, the majority of participants (56·7 %), is characterised by a normal weight on average but increasing. Group 2, around one-third (34·7 %), is characterised by being overweight on average and this is increasing with time. Group 3, the trajectory with the smallest proportion of participants (8·6 %), is characterised by the mean BMI being classified as obese throughout the period and the mean BMI is increasing faster than the other groups. There was a good model fit with an average posterior probability of assignment for each group higher than 0·7, the odds of correct classification were higher than 5·0, and the observed *v*. estimated proportion assigned to each group was similar.

The characteristics of participants in each trajectory group is presented in Table 2. Compared with other trajectory groups, participants in group 1 have a higher proportion of males than females. Participants born in 1970s to 1990s comprise the highest proportion in all groups.

**Table 2:** The characteristics of people in trajectory each group

	Group 1		Group 2		Group 3	
	*n* 24 641		*n* 14 382		*n* 3514	
	*n*	%	*n*	%	*n*	%
Sex						
Male	13 583	55	6160	43	1035	30
Female	11 058	45	8222	57	2479	70
Birth cohort						
Born 1930s and before	2844	12	911	6	258	7
Born 1940s	1699	7	972	7	327	9
Born 1950s	2061	8	1805	13	671	19
Born 1960s	3002	12	2899	20	757	22
Born 1970—1990s	15 035	61	7795	54	1501	43

**Fig. 5:**
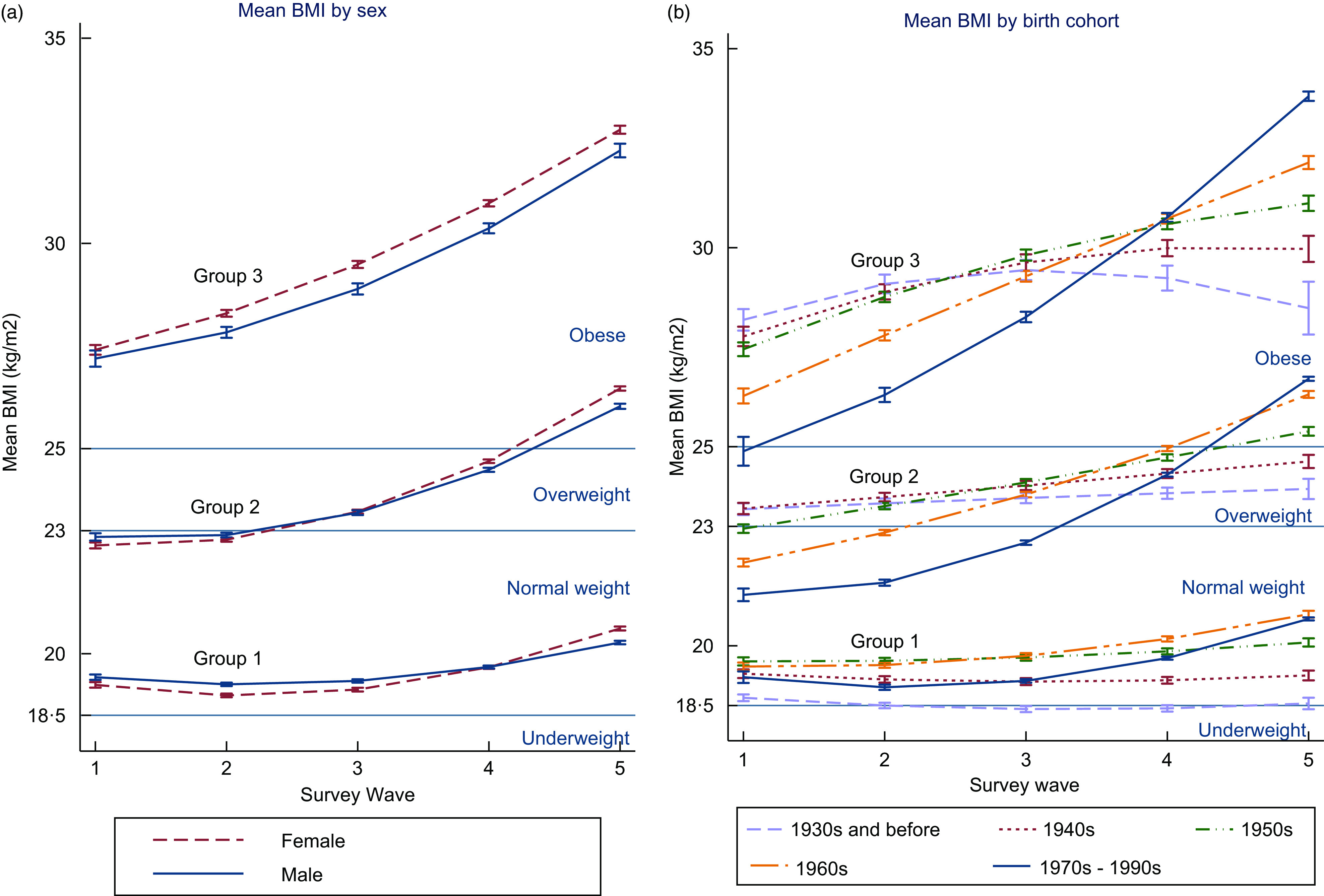
Mean BMI per trajectory groups, by sex and birth cohort: (a) Mean BMI per trajectory groups by sex. (b) Mean BMI per trajectory groups by birth cohort

Using mixed models, we found that these trajectories were different by both sex (*P* < 0·001) and birth cohort (*P* < 0·001). Figure 5 displays the interaction effects for BMI by sex (a) and birth cohort (b). Graph (a) (left) shows the differences in mean BMI between males and females. Among groups 1 and 2, in 1993, males had a higher mean BMI than females, but this was reversed by the last survey wave, with females having a higher mean BMI in 2014. Among group 3, females had a higher mean BMI throughout the entire study period. Graph (b) (right) shows the pattern of mean BMI among different birth cohorts for each of the three trajectory groups. The mean BMI of the youngest cohorts (born in 1970–1990s) increased faster over time in all three trajectory groups compared to older birth cohorts (born 1940–1960s). In contrast, the mean BMI in the oldest birth cohort (born in 1930s and earlier) remained stable over time or showed a slight decrease among group 1.

## Discussion

This study demonstrates mean BMI has increased among Indonesian adults between 1993 and 2014. Longitudinal analysis shows that the increase in mean BMI over the period is being driven by younger cohorts (born in 1970—1990s) who have a higher mean BMI and a greater increase in BMI over time than those from older cohorts. There are also differences by sex, with females having a higher mean BMI than males in the latest survey wave. Using group-based trajectory methods, we identified three groups. All three groups have quadratic increases in mean BMI over time, with mean BMI of group 3 increasing more than groups 1 and 2 (Fig. 4). The greatest increase in mean BMI for all three groups has occurred since 2007. Group 3 (representing 8·6 % of the participants) shows the greatest increase in BMI over the period 1993 to 2014 with mean BMI increasing from 27·1 kg/m^2^ to 32·6 kg/m^2^.

Three previous studies examining BMI among Indonesian adults have also found an increased prevalence of overweight and obesity over time; however, these have been based on repeated cross-sectional analysis^(8,27,28)^. Oddo et al. (2019) reported a two-fold increase in overweight prevalence from 17·1 % in 1993 to 33 % in 2014 based on analysis of the IFLS as five repeated cross-sectional surveys rather than longitudinal analysis^(9)^. Another study using four cross-sectional surveys of the IFLS up until 2007 reported that obesity prevalence increased two-fold both in males (4·1 % in 1993 to 8·9 % in 2007) and females (9·7 % in 1993 to 19·6 % in 2007)^(7)^. Using the same the IFLS data, Vaezghasemi et al. (2016) reported that the mean BMI of adults increased from 21·5 kg/m^2^ in 1993 to 22·8 kg/m^2^ in 2007^(8)^. All of these studies showed that females have higher overweight and obesity prevalence as well as higher mean BMI compared to males.

The findings of the current study are also in line with the current trend of overweight and obesity seen in many low-middle income countries in the Pacific, the Caribbean, the Middle East, Central America and South East Asia^(2,5)^. These studies suggest that obesity prevalence is now increasing at a faster rate in low-middle income countries than in high-income countries, although these increases started earlier in high-income countries ^(5)^. This study confirms that this is occurring in Indonesia^(6,29)^.

Studies examining trajectories of BMI using longitudinal study designs in low-middle income countries setting are scarce. A study in China using longitudinal data found four distinct trajectories of weight change with a quadratic effect among the overweight to obese group^(30)^. There are however several studies that have examined BMI trajectories using longitudinal data in high-income countries. Three of these studies from USA, Finland and Australia reported that BMI increased with age from early adulthood into middle age and tended to decrease at older ages^(31–33)^. Similarly, the current study also portrayed complex patterns in longitudinal data sets by revealing three distinctive development trajectories of BMI in population. Indonesia is currently experiencing a Double Burden of Malnutrition, indicated by a high prevalence of under-nutrition problems such as underweight, stunting and wasting in conjunction with overweight and obesity issues^(34)^. We believe that BMI change in Indonesia cannot be fully described by a single parameter. It is crucial to take the variance in the population into account in order to prioritise obesity interventions targeted to high-risk groups.

Another important finding from this study was that individuals who were already obese at the beginning of the survey tended to increase their BMI much faster over time than those who started from a normal weight. This suggests that the prevalence of overweight and obesity in Indonesia will continue to increase and potentially lead to increases in non-communicable disease prevalence if major prevention and intervention actions are not taken^(35)^.

This is the first study observing trends of BMI in Indonesia adults examining different patterns of trajectories by sex and birth cohort. Strengths of the current study include the use of longitudinal analyses from a large population-based longitudinal survey covering a 21-year period, with more than 90 % re-contact rates at each wave (65 % of participants have two or more measures). Therefore, the dynamic and individual differences in growth rate can be captured through different patterns in BMI trajectory^(36)^. The longitudinal nature allows us to investigate how individual people change over time^(37)^. A further strength is that the anthropometry assessments were taken by trained health workers rather than by self-report. There were some limitations; not all individuals participated in all five waves of data collection, leading to missing data and loss of follow-up^(38)^. However, the number lost to follow-up was relatively small (less than 10 % per wave). Moreover, the use of mixed model analysis allows us to include all eligible participants regardless of how many times they participated in the study^(23,24)^. BMI is a widely used measure in population-based epidemiological studies; however, there are limitations to BMI as a useful measure in some individuals and population groups^(39)^. BMI does not distinguish the origin of body weight (e.g. from muscle mass or fat mass) as it is a measure of body size rather than composition. Accurate assessment of body composition in individuals involves more complex and expensive tools such as dual-energy X-ray absorptiometry^(40)^.

In conclusion, this study provides robust new evidence on the trajectories of BMI among Indonesian adults. The mean BMI patterns confirm that Indonesia is in the process of a rapid and intense nutrition transition^(41)^. Both males and females show increased BMI over the period on average. However, the more recent generations show greater increases in mean BMI than the earlier generations. The potential implications of the positive quadratic effect (with greater increases in mean BMI over the period from last three surveys) indicate an accelerated increase in BMI that is alarming in relation to chronic disease risk and the potential impact on public health and health services.

Further research using the same and/or other datasets to examine risk factors of overweight/obesity and different population such as children and adolescents are needed to continue to gain a better understanding of the epidemiology of overweight and obesity in the Indonesian population.

## Supplementary material

For supplementary material accompanying this paper visit https://doi.org/10.1017/S1368980023000472. click here to view supplementary material
