# Supplementary material for: Trends in BMI of Indonesian adults between 1993 and 2014: a longitudinal population-based study
Source: Public Health Nutr. 2023 Mar 13;26(7):1394–402. doi: 10.1017/S1368980023000472 (PMC10346025; doi:10.1017/S1368980023000472)
Supplement: Supplementary file 1 [file S1368980023000472sup001.docx]

Supplementary Material

Table 1 Mean BMI in adults by gender and year of survey

| Survey year | All gender | | Male | | Female | |
| --- | --- | --- | --- | --- | --- | --- |
|  | n | Mean(SD) | n | Mean(SD) | n | Mean(SD) |
| 1993 | 12,837 | 21.4 (3.4) | 5,683 | 21.0 (3.0) | 7,154 | 21.7 (3.7) |
| 1997 | 16,653 | 21.6 (3.6) | 7,448 | 21.1 (3.1) | 9,205 | 22.0 (3.9) |
| 2000 | 21,304 | 21.7 (3.7) | 10,261 | 21.2 (3.2) | 11,043 | 22.2 (4.1) |
| 2007 | 25,954 | 22.5 (4.1) | 12,460 | 21.8 (3.6) | 13,494 | 23.2 (4.4) |
| 2014 | 29,072 | 23.5 (4.5) | 13,832 | 22.5 (3.9) | 15,240 | 24.4 (4.8) |
| *Combined* | *105,820* | *22.3* | *49,684* | *21.7 (3.5)* | *56,136* | *22.9 (4.4)* |

Table 2 Mean BMI of adults, by age group and year of survey, 1993 – 2014

| Age category (years) | 1993 | | 1997 | | 2000 | | 2007 | | 2014 | |
| --- | --- | --- | --- | --- | --- | --- | --- | --- | --- | --- |
|  | n | Mean(SD) | n | Mean(SD) | n | Mean(SD) | n | Mean(SD) | n | Mean(SD) |
| BMI all genders | | | | | | | | | | |
| 19 – 24 | 955 | 20.8(2.6) | 2,579 | 20.7(2.8) | 3,938 | 20.6(3.0) | 4,064 | 21.1(3.4) | 3,837 | 21.7(4.0) |
| 25 – 29 | 1,496 | 21.5(2.8) | 2,048 | 21.4(3.0) | 2,889 | 21.5(3.2) | 4,156 | 22.1(3.8) | 3,715 | 23.1(4.4) |
| 30 – 34 | 1,870 | 21.8(3.1) | 2,197 | 22.1(3.3) | 2,668 | 22.2(3.6) | 3,457 | 22.9(3.9) | 4,443 | 23.7(4.4) |
| 35 – 39 | 1,751 | 22.1(3.4) | 2,025 | 22.4(3.5) | 2,436 | 22.7(3.6) | 3,082 | 23.5(4.1) | 3,862 | 24.3(4.4) |
| 40 – 44 | 1,425 | 22.2(3.6) | 1,725 | 22.9(3.9) | 2,135 | 22.9(3.9) | 2,483 | 23.8(4.2) | 3,108 | 24.6(4.5) |
| 45 – 49 | 1,020 | 21.8(3.6) | 1,349 | 22.4(3.9) | 1,754 | 23.0(4.1) | 2,240 | 23.6(4.3) | 2,571 | 24.6(4.4) |
| 50 – 54 | 1,268 | 21.3(3.7) | 1,150 | 21.7(3.8) | 1,206 | 22.5(3.9) | 1,767 | 23.5(4.3) | 2,182 | 24.4(4.5) |
| 55 – 59 | 906 | 20.9(3.6) | 1,171 | 21.3(3.8) | 1,272 | 21.6(4.1) | 1,332 | 22.9(4.3) | 1,714 | 23.9(4.5) |
| 60 – 64 | 839 | 20.5(3.7) | 881 | 20.8(3.8) | 998 | 21.0(3.9) | 991 | 22.1(4.3) | 1,326 | 23.3(4.5) |
| 65 – 69 | 526 | 20.3(3.6) | 636 | 20.7(4.3) | 786 | 20.7(4.2) | 988 | 21.3(4.2) | 820 | 22.6(4.1) |
| 70 – 75 | 407 | 19.6(3.6) | 439 | 20.0(3.6) | 589 | 20.2(3.7) | 605 | 20.7(3.9) | 741 | 21.3(4.0) |
| 75+ | 374 | 19.6(3.3) | 453 | 19.2(3.3) | 632 | 19.2(3.4) | 789 | 20.2(3.5) | 753 | 20.3(3.5) |
| *Total* | *12,837* |  | *16,653* |  | *21,304* |  | *25,954* |  | *29,072* |  |
| BMI male |  |  |  |  |  |  |  |  |  |  |
| 19 – 24 | 286 | 20.4(2.0) | 1,167 | 20.3(2.5) | 1,946 | 20.3(2.7) | 1,863 | 20.6(3.0) | 1,755 | 21.1(3.7) |
| 25 – 29 | 576 | 20.9(2.4) | 878 | 21.0(2.7) | 1,461 | 21.0(3.0) | 2,019 | 21.4(3.4) | 1,730 | 22.1(3.9) |
| 30 – 34 | 809 | 21.3(2.6) | 937 | 21.5(2.9) | 1,331 | 21.6(3.1) | 1,756 | 22.1(3.5) | 2,134 | 22.5(3.9) |
| 35 – 39 | 811 | 21.6(2.9) | 897 | 21.7(2.9) | 1,132 | 21.8(3.2) | 1,579 | 22.7(3.6) | 1,912 | 23.1(3.9) |
| 40 – 44 | 695 | 21.8(3.2) | 760 | 22.0(3.3) | 1,013 | 22.1(3.2) | 1,198 | 22.8(3.7) | 1,569 | 23.4(3.8) |
| 45 – 49 | 509 | 21.6(3.2) | 671 | 21.7(3.2) | 844 | 22.2(3.5) | 1,054 | 22.8(3.7) | 1,262 | 23.6(3.9) |
| 50 – 54 | 536 | 20.9(3.2) | 499 | 21.4(3.4) | 594 | 21.9(3.4) | 824 | 22.7(3.7) | 987 | 23.4(3.9) |
| 55 – 59 | 435 | 20.7(3.2) | 522 | 20.9(3.1) | 564 | 21.1(3.5) | 655 | 22.2(3.6) | 803 | 22.9(3.9) |
| 60 – 64 | 383 | 20.2(3.1) | 394 | 20.4(3.0) | 457 | 20.7(3.2) | 455 | 21.5(3.6) | 621 | 22.3(3.7) |
| 65 – 69 | 276 | 20.1(3.3) | 305 | 20.1(3.3) | 356 | 20.1(3.1) | 444 | 20.9(3.4) | 395 | 21.8(3.7) |
| 70 – 74 | 198 | 19.1(2.6) | 209 | 19.7(3.1) | 280 | 19.8(3.2) | 268 | 20.2(3.2) | 331 | 20.6(3.5) |
| 75+ | 169 | 19.2(3.0) | 209 | 18.9(2.7) | 283 | 18.8(2.7) | 345 | 19.7(3.1) | 333 | 20.1(3.1) |
| *Total* | *5,683* |  | *7,448* |  | *10,261* |  | *12,460* |  | *13,832* |  |
| BMI female |  |  |  |  |  |  |  |  |  |  |
| 19 – 24 | 669 | 20.9(2.8) | 1,412 | 21.0(3.0) | 1,992 | 20.9(3.2) | 2,201 | 21.5(3.7) | 2,082 | 22.3(4.3) |
| 25 – 29 | 920 | 21.8(3.0) | 1,170 | 21.7(3.2) | 1,428 | 21.9(3.4) | 2,137 | 22.8(4.0) | 1,985 | 24.0(4.6) |
| 30 – 34 | 1,061 | 22.2(3.5) | 1,260 | 22.6(3.4) | 1,337 | 22.7(3.8) | 1,701 | 23.8(4.2) | 2,309 | 24.8(4.6) |
| 35 – 39 | 940 | 22.7(3.7) | 1,128 | 23.0(3.9) | 1,304 | 23.4(3.8) | 1,503 | 24.3(4.4) | 1,950 | 25.4(4.6) |
| 40 – 44 | 730 | 22.7(3.9) | 965 | 23.5(4.2) | 1,122 | 23.6(4.3) | 1,285 | 24.6(4.3) | 1,539 | 25.8(4.8) |
| 45 – 49 | 511 | 22.1(3.9) | 678 | 23.2(4.4) | 910 | 23.7(4.5) | 1,186 | 24.4(4.6) | 1,309 | 25.7(4.5) |
| 50 – 54 | 732 | 21.5(4.0) | 651 | 22.0(4.0) | 612 | 23.0(4.3) | 943 | 24.3(4.7) | 1,195 | 25.2(4.8) |
| 55 – 59 | 471 | 21.1(3.9) | 649 | 21.6(4.2) | 708 | 22.0(4.6) | 677 | 23.7(4.8) | 911 | 24.8(4.8) |
| 60 – 64 | 456 | 20.7(4.1) | 487 | 21.1(4.2) | 541 | 21.2(4.4) | 536 | 22.7(4.7) | 705 | 24.2(4.9) |
| 65 – 69 | 250 | 20.5(3.9) | 331 | 21.3(4.9) | 430 | 21.1(4.8) | 544 | 21.7(4.8) | 425 | 23.3(4.4) |
| 70 – 74 | 209 | 20.0(4.3) | 230 | 20.2(3.9) | 309 | 20.7(4.1) | 337 | 21.0(4.3) | 410 | 21.8(4.3) |
| 75+ | 205 | 19.8(3.5) | 244 | 19.5(3.8) | 349 | 19.5(3.9) | 444 | 20.5(38) | 420 | 20.5(3.8) |
| *Total* | *7,154* |  | *9,205* |  | *11,043* |  | *13,494* |  | *15,240* |  |

Table 3 Mean BMI of adults, by year of survey for different age groups

| Survey year | 19-29 | | 30-39 | | 40-49 | | 50-59 | | 60-69 | | 70+ | |
| --- | --- | --- | --- | --- | --- | --- | --- | --- | --- | --- | --- | --- |
|  | n | Mean(SD) | n | Mean(SD) | n | Mean(SD) | n | Mean(SD) | n | Mean(SD) | n | Mean(SD) |
| All genders | | | | | | | | | | | | |
| 1993 | 2,451 | 21.2 (2.7) | 3,621 | 22.0 (3.3) | 2,445 | 22.1 (3.6) | 2,174 | 21.1 (3.7) | 1,365 | 20.4 (3.7) | 781 | 19.6 (3.4) |
| 1997 | 4,627 | 21.0 (2.9) | 4,222 | 22.2 (3.4) | 3,074 | 22.7 (3.9) | 2,321 | 21.5 (3.8) | 1,517 | 20.7 (4.0) | 892 | 19.6 (3.5) |
| 2000 | 6,827 | 21.0 (3.1) | 5,104 | 22.4 (3.6) | 3,889 | 22.9 (4.0) | 2,478 | 22.0 (4.0) | 1,784 | 20.8 (4.0) | 1,222 | 19.7 (3.6) |
| 2007 | 8,220 | 21.6 (3.6) | 6,539 | 23.2 (4.0) | 4,723 | 23.7 (4.2) | 3,099 | 23.3 (4.3) | 1,979 | 21.7 (4.3) | 1,394 | 20.4 (3.7) |
| 2014 | 7,552 | 22.4 (4.3) | 8,305 | 24.0 (4.4) | 5,679 | 24.6 (4.4) | 3,896 | 24.2 (4.5) | 2,146 | 23.0 (4.3) | 1,494 | 20.8 (3.8) |
| *Total* | *29,677* |  | *27,791* |  | *19,810* |  | *13,968* |  | *8,791* |  | *5,783* |  |
| Male |  |  |  |  |  |  |  |  |  |  |  |  |
| 1993 | 862 | 20.8 (2.3) | 1,620 | 21.4 (2.7) | 1,204 | 21.7 (3.2) | 971 | 20.8 (3.2) | 659 | 20.1 (3.2) | 367 | 19.2 (2.8) |
| 1997 | 2,045 | 20.6 (2.6) | 1,834 | 21.6 (2.9) | 1,431 | 21.9 (3.2) | 1,021 | 21.1 (3.3) | 699 | 20.2 (3.2) | 418 | 19.3 (2.9) |
| 2000 | 3,407 | 20.6 (2.8) | 2,463 | 21.7 (3.2) | 1,857 | 22.1 (3.4) | 1,158 | 21.5 (3.4) | 813 | 20.4 (3.2) | 563 | 19.3 (3.0) |
| 2007 | 3,882 | 21.0 (3.2) | 3,335 | 22.4 (3.5) | 2,252 | 22.8 (3.7) | 1,479 | 22.4 (3.7) | 899 | 21.2 (3.5) | 613 | 20.0 (3.2) |
| 2014 | 3,485 | 21.6 (3.8) | 4,046 | 22.8 (3.9) | 2,831 | 23.5 (3.9) | 1,790 | 23.2 (3.9) | 1,016 | 22.1 (3.7) | 664 | 20.4 (3.3) |
| *Total* | *13,681* |  | *13,298* |  | *9,575* |  | *6,419* |  | *4,086* |  | *2,625* |  |
| Female |  |  |  |  |  |  |  |  |  |  |  |  |
| 1993 | 1,589 | 21.4 (2.9) | 2,001 | 22.4 (3.6) | 1,241 | 22.4 (3.9) | 1,203 | 21.4 (4.0) | 706 | 20.7 (4.0) | 414 | 19.9 (3.9) |
| 1997 | 2,582 | 21.4 (3.1) | 2,388 | 22.8 (3.7) | 1,643 | 23.4 (4.3) | 1,300 | 21.8 (4.2) | 818 | 21.2 (4.5) | 474 | 19.8 (3.9) |
| 2000 | 3,420 | 21.3 (3.3) | 2,641 | 23.1 (3.9) | 2,032 | 23.6 (4.4) | 1,320 | 22.4 (4.5) | 971 | 21.2 (4.6) | 659 | 20.0 (4.0) |
| 2007 | 4,338 | 22.1 (3.9) | 3,204 | 24.0 (4.3) | 2,471 | 24.5 (4.5) | 1,620 | 24.0 (4.7) | 1,080 | 22.2 (4.8) | 781 | 20.7 (4.0) |
| 2014 | 4,067 | 23.1 (4.5) | 4,259 | 25.1 (4.6) | 2,848 | 25.7 (4.7) | 2,106 | 25.0 (4.8) | 1,130 | 23.9 (4.7) | 830 | 21.1 (4.1) |
| *Total* | *15,996* |  | *14,493* |  | *10,235* |  | *7,549* |  | *4,705* |  | *3,158* |  |

Table 4 Mean BMI of adults, by age when measured for different birth cohort

|  | Born before 1930s | | Born 1930s | | Born 1940s | | Born 1950s | | Born 1960s | | Born 1970s | | Born 1980s | | Born 1990s | |
| --- | --- | --- | --- | --- | --- | --- | --- | --- | --- | --- | --- | --- | --- | --- | --- | --- |
|  | n | mean(SD) | n | mean(SD) | n | mean(SD) | n | mean(SD) | n | mean(SD) | n | mean(SD) | n | mean(SD) | n | mean(SD) |
| BMI all |  |  |  |  |  |  |  |  |  |  |  |  |  |  |  |  |
| 19 – 24 | 0 | 0 | 0 | 0 | 0 | 0 | 0 | 0 | 204 | 20.6(2.4) | 5,859 | 20.7(2.8) | 5,473 | 20.9(3.3) | 3,837 | 21.7(4.0) |
| 24 – 29 | 0 | 0 | 0 | 0 | 0 | 0 | 0 | 0 | 2,345 | 21.5(3.0) | 5,707 | 21.7(3.4) | 6,252 | 22.6(4.2) | 0 | 0 |
| 30 – 34 | 0 | 0 | 0 | 0 | 0 | 0 | 343 | 22.1(3.3) | 5,814 | 22.1(3.3) | 4,035 | 22.8(3.9) | 4,443 | 23.7(4.4) | 0 | 0 |
| 35 – 39 | 0 | 0 | 0 | 0 | 0 | 0 | 2,612 | 22.2(3.5) | 4,806 | 22.9(3.8) | 5,738 | 24.0(4.3) | 0 | 0 | 0 | 0 |
| 40 – 44 | 0 | 0 | 0 | 0 | 208 | 22.3(3.4) | 4,619 | 22.7(3.9) | 2,941 | 23.6(4.1) | 3,108 | 24.6(4.5) | 0 | 0 | 0 | 0 |
| 45 – 49 | 0 | 0 | 0 | 0 | 1,518 | 21.9(3.6) | 3,468 | 23.0(4.2) | 3,948 | 24.3(4.3) | 0 | 0 | 0 | 0 | 0 | 0 |
| 50 – 54 | 0 | 0 | 182 | 21.3(3.7) | 3,164 | 21.8(3.8) | 2,045 | 23.4(4.2) | 2,182 | 24.4(4.5) | 0 | 0 | 0 | 0 | 0 | 0 |
| 55 – 59 | 0 | 0 | 1,342 | 21.0(3.6) | 2,461 | 21.7(4.1) | 2,592 | 23.7(4.5) | 0 | 0 | 0 | 0 | 0 | 0 | 0 | 0 |
| 60 – 64 | 90 | 21.2(4.1) | 2,384 | 20.7(3.8) | 1,235 | 21.9(4.3) | 1,326 | 23.3(4.5) | 0 | 0 | 0 | 0 | 0 | 0 | 0 | 0 |
| 65 – 69 | 759 | 20.3(3.7) | 1,525 | 20.9(4.3) | 1,472 | 22.1(4.2) | 0 | 0 | 0 | 0 | 0 | 0 | 0 | 0 | 0 | 0 |
| 70 – 74 | 1,260 | 20.0(3.6) | 780 | 20.5(3.8) | 741 | 21.3(4.0) | 0 | 0 | 0 | 0 | 0 | 0 | 0 | 0 | 0 | 0 |
| 75+ | 2,130 | 19.5(3.3) | 872 | 20.5(3.6) | 0 | 0 | 0 | 0 | 0 | 0 | 0 | 0 | 0 | 0 | 0 | 0 |
| *Total* | *4,239* |  | *7,085* |  | *10,799* |  | *17,005* |  | *22,240* |  | *24,447* |  | *16,168* |  | *3,837* |  |
| BMI male |  |  |  |  |  |  |  |  |  |  |  |  |  |  |  |  |
| 19 – 24 1 | 0 | 0 | 0 | 0 | 0 | 0 | 0 | 0 | 83 | 20.5(2.2) | 2,616 | 20.4(2.6) | 2,563 | 20.4(2.9) | 1,755 | 21.1(3.7) |
| 25 – 29 2 | 0 | 0 | 0 | 0 | 0 | 0 | 0 | 0 | 928 | 21.0(2.6) | 2,790 | 21.2(3.1) | 2,946 | 21.7(3.6) | 0 | 0 |
| 30 – 34 3 | 0 | 0 | 0 | 0 | 0 | 0 | 154 | 21.4(2.8) | 2,622 | 21.5(2.9) | 2,057 | 22.0(3.4) | 2,134 | 22.5(3.9) | 0 | 0 |
| 35 – 39 4 | 0 | 0 | 0 | 0 | 0 | 0 | 1,218 | 21.6(3.0) | 2,214 | 22.1(3.2) | 2,899 | 23.0(3.8) | 0 | 0 | 0 | 0 |
| 40 – 44 5 | 0 | 0 | 0 | 0 | 109 | 21.7(3.2) | 2,139 | 22.0(3.3) | 1,418 | 22.7(3.6) | 1,569 | 23.4(3.8) | 0 | 0 | 0 | 0 |
| 45 – 49 6 | 0 | 0 | 0 | 0 | 752 | 21.6(3.1) | 1,697 | 22.2(3.5) | 1,891 | 23.3(3.9) | 0 | 0 | 0 | 0 | 0 | 0 |
| 50 – 54 7 | 0 | 0 | 88 | 21.1(3.4) | 1,394 | 21.4(3.4) | 971 | 22.5(3.6) | 987 | 23.4(3.9) | 0 | 0 | 0 | 0 | 0 | 0 |
| 55 – 59 8 | 0 | 0 | 637 | 20.8(3.2) | 1,105 | 21.2(3.4) | 1,237 | 22.7(3.8) | 0 | 0 | 0 | 0 | 0 | 0 | 0 | 0 |
| 60 – 64 9 | 52 | 20.3(3.5) | 1,083 | 20.4(3.1) | 554 | 21.3(3.6) | 621 | 22.3(3.7) | 0 | 0 | 0 | 0 | 0 | 0 | 0 | 0 |
| 65 – 69 10 | 391 | 20.0(3.4) | 705 | 20.4(3.3) | 680 | 21.3(3.6) | 0 | 0 | 0 | 0 | 0 | 0 | 0 | 0 | 0 | 0 |
| 70 – 74 11 | 614 | 19.5(3.0) | 341 | 20.1(3.2) | 331 | 20.6(3.5) | 0 | 0 | 0 | 0 | 0 | 0 | 0 | 0 | 0 | 0 |
| 75+ 12 | 964 | 19.1(2.8) | 375 | 20.2(3.2) | 0 | 0 | 0 | 0 | 0 | 0 | 0 | 0 | 0 | 0 | 0 | 0 |
| *Total* | *2,021* |  | *3,229* |  | *4,925* |  | *8,037* |  | *10,143* |  | *11,931* |  | *7,643* |  | *1,755* |  |
| BMI female |  |  |  |  |  |  |  |  |  |  |  |  |  |  |  |  |
| 19 – 24 1 | 0 | 0 | 0 | 0 | 0 | 0 | 0 | 0 | 121 | 20.7(2.6) | 3,243 | 21.0(3.0) | 2,910 | 21.3(3.6) | 2,082 | 22.3(4.3) |
| 25 – 29 2 | 0 | 0 | 0 | 0 | 0 | 0 | 0 | 0 | 1,417 | 21.9(3.1) | 2,917 | 22.1(3.5) | 3,306 | 23.4(4.4) | 0 | 0 |
| 30 – 34 3 | 0 | 0 | 0 | 0 | 0 | 0 | 189 | 22.6(3.6) | 3,192 | 22.5(3.6) | 1,978 | 23.6(4.2) | 2,309 | 24.8(4.6) | 0 | 0 |
| 35 – 39 4 | 0 | 0 | 0 | 0 | 0 | 0 | 1,394 | 22.8(3.8) | 2,592 | 23.6(4.0) | 2,839 | 25.0(4.5) | 0 | 0 | 0 | 0 |
| 40 – 44 5 | 0 | 0 | 0 | 0 | 99 | 23.0(3.5) | 2,480 | 23.4(4.2) | 1,523 | 24.4(4.3) | 1,539 | 25.8(4.8) | 0 | 0 | 0 | 0 |
| 45 – 49 6 | 0 | 0 | 0 | 0 | 766 | 22.2(3.9) | 1,771 | 23.8(4.6) | 2,057 | 25.2(4.6) | 0 | 0 | 0 | 0 | 0 | 0 |
| 50 – 54 7 | 0 | 0 | 94 | 21.6(4.1) | 1,770 | 22.0(4.1) | 1,074 | 24.2(4.6) | 1,195 | 25.2(4.8) | 0 | 0 | 0 | 0 | 0 | 0 |
| 55 – 59 8 | 0 | 0 | 705 | 21.2(4.0) | 1,356 | 22.1(4.5) | 1,355 | 24.5(4.8) | 0 | 0 | 0 | 0 | 0 | 0 | 0 | 0 |
| 60 – 64 9 | 38 | 22.3(4.6) | 1,301 | 21.0(4.2) | 681 | 22.4(4.7) | 705 | 24.2(4.9) | 0 | 0 | 0 | 0 | 0 | 0 | 0 | 0 |
| 65 – 69 10 | 368 | 20.6(4.0) | 820 | 21.3(4.9) | 792 | 22.7(4.6) | 0 | 0 | 0 | 0 | 0 | 0 | 0 | 0 | 0 | 0 |
| 70 – 74 11 | 646 | 20.4(4.1) | 439 | 20.8(4.2) | 410 | 21.8(4.3) | 0 | 0 | 0 | 0 | 0 | 0 | 0 | 0 | 0 | 0 |
| 75+ 12 | 1,166 | 19.8(3.7) | 497 | 20.7(3.9) | 0 | 0 | 0 | 0 | 0 | 0 | 0 | 0 | 0 | 0 | 0 | 0 |
| *Total* | *2,218* |  | *3,856* |  | *5,874* |  | *8,968* |  | *12,097* |  | *12,516* |  | *8,525* |  | *2,082* |  |

Table 5 Mean BMI of adults, by birth cohort for different age groups

| Year of birth | 19-29 | | 30-39 | | 40-49 | | 50-59 | | 60-69 | | 70+ | |
| --- | --- | --- | --- | --- | --- | --- | --- | --- | --- | --- | --- | --- |
|  | n | Mean(SD) | n | Mean(SD) | n | Mean(SD) | n | Mean(SD) | n | Mean(SD) | n | Mean(SD) |
| All genders | | | | | | | | | | | | |
| < 1930s | 0 | 0 | 0 | 0 | 0 | 0 | 0 | 0 | 849 | 20.4 (3.7) | 3,390 | 19.7 (3.5) |
| 1930s | 0 | 0 | 0 | 0 | 0 | 0 | 1,524 | 21.0 (3.6) | 3,909 | 20.8 (4.0) | 1,652 | 20.5 (3.7) |
| 1940s | 0 | 0 | 0 | 0 | 1,726 | 21.9 (3.6) | 5,625 | 21.7 (3.9) | 2,707 | 22.0 (4.2) | 741 | 21.3 (4.0) |
| 1950s | 0 | 0 | 2,955 | 22.2 (3.5) | 8,087 | 22.9 (4.0) | 4,637 | 23.6 (4.4) | 1,326 | 23.3 (4.5) | 0 | 0 |
| 1960s | 2,549 | 21.5 (2.9) | 10,620 | 22.4 (3.6) | 6,889 | 24.0 (4.3) | 2,182 | 24.4 (4.5) | 0 | 0 | 0 | 0 |
| 1970s | 11,566 | 21.2 (3.1) | 9,773 | 23.5 (4.2) | 3,108 | 24.6 (4.5) | 0 | 0 | 0 | 0 | 0 | 0 |
| 1980s | 11,725 | 21.8 (3.9) | 4,443 | 23.7 (4.4) | 0 | 0 | 0 | 0 | 0 | 0 | 0 | 0 |
| 1990s | 3,837 | 21.7 (4.0) | 0 | 0 | 0 | 0 | 0 | 0 | 0 | 0 | 0 | 0 |
| *Total* | *29,677* |  | *27,791* |  | *19,810* |  | *13,968* |  | *8,791* |  | *5,783* |  |
| Male |  |  |  |  |  |  |  |  |  |  |  |  |
| < 1930s | 0 | 0 | 0 | 0 | 0 | 0 | 0 | 0 | 443 | 20.1 (3.4) | 1,578 | 19.3 (2.9) |
| 1930s | 0 | 0 | 0 | 0 | 0 | 0 | 725 | 20.8 (3.2) | 1,788 | 20.4 (3.2) | 716 | 20.1 (3.2) |
| 1940s | 0 | 0 | 0 | 0 | 861 | 21.6 (3.1) | 2,499 | 21.3 (3.4) | 1,234 | 21.3 (3.6) | 331 | 20.6 (3.5) |
| 1950s | 0 | 0 | 1,372 | 21.6 (3.0) | 3,836 | 22.1 (3.4) | 2,208 | 22.6 (3.7) | 621 | 22.3 (3.7) | 0 | 0 |
| 1960s | 1,011 | 21.0 (2.5) | 4,836 | 21.8 (3.1) | 3,309 | 23.1 (3.8) | 987 | 23.4 (3.9) | 0 | 0 | 0 | 0 |
| 1970s | 5,406 | 20.8 (2.9) | 4,956 | 22.6 (3.7) | 1,569 | 23.4 (3.8) | 0 | 0 | 0 | 0 | 0 | 0 |
| 1980s | 5,509 | 21.1 (3.4) | 2,134 | 22.5 (3.9) | 0 | 0 | 0 | 0 | 0 | 0 | 0 | 0 |
| 1990s | 1,755 | 21.1 (3.7) | 0 | 0 | 0 | 0 | 0 | 0 | 0 | 0 | 0 | 0 |
| *Total* | *13,681* |  | *13,298* |  | *9,575* |  | *6,419* |  | *4,086* |  | *2,625* |  |
| Female |  |  |  |  |  |  |  |  |  |  |  |  |
| < 1930s | 0 | 0 | 0 | 0 | 0 | 0 | 0 | 0 | 406 | 20.8 (4.1) | 1,812 | 20.0 (3.9) |
| 1930s | 0 | 0 | 0 | 0 | 0 | 0 | 799 | 21.2 (4.0) | 2,121 | 21.1 (4.5) | 936 | 20.8 (4.1) |
| 1940s | 0 | 0 | 0 | 0 | 865 | 22.3 (3.9) | 3,126 | 22.1 (4.3) | 1,473 | 22.6 (4.6) | 410 | 21.8 (4.3) |
| 1950s | 0 | 0 | 1,583 | 22.8 (3.8) | 4,251 | 23.5 (4.4) | 2,429 | 24.4 (4.7) | 705 | 24.2 (4.9) | 0 | 0 |
| 1960s | 1,538 | 21.8 (3.1) | 5,784 | 23.0 (3.8) | 3,580 | 24.9 (4.5) | 1,195 | 25.2 (4.8) | 0 | 0 | 0 | 0 |
| 1970s | 6,160 | 21.5 (3.3) | 4,817 | 24.4 (4.4) | 1,539 | 25.8 (4.8) | 0 | 0 | 0 | 0 | 0 | 0 |
| 1980s | 6,216 | 22.4 (4.2) | 2,309 | 24.8 (4.6) | 0 | 0 | 0 | 0 | 0 | 0 | 0 | 0 |
| 1990s | 2,082 | 22.3 (4.3) | 0 | 0 | 0 | 0 | 0 | 0 | 0 | 0 | 0 | 0 |
| *Total* | *15,996* |  | *14,493* |  | *10,235* |  | *7,549* |  | *4,705* |  | *3,158* |  |
